# Inhibition of *Postn* Rescues Myogenesis Defects in Myotonic Dystrophy Type 1 Myoblast Model

**DOI:** 10.3389/fcell.2021.710112

**Published:** 2021-08-19

**Authors:** Xiaopeng Shen, Zhongxian Liu, Chunguang Wang, Feng Xu, Jingyi Zhang, Meng Li, Yang Lei, Ao Wang, Chao Bi, Guoping Zhu

**Affiliations:** ^1^Anhui Provincial Key Laboratory of Molecular Enzymology and Mechanism of Major Diseases, College of Life Sciences, Anhui Normal University, Wuhu, China; ^2^Anhui Provincial Key Laboratory of the Conservation and Exploitation of Biological Resources, College of Life Sciences, Anhui Normal University, Wuhu, China; ^3^Key Laboratory of Biomedicine in Gene Diseases and Health of Anhui Higher Education Institutes, College of Life Sciences, Anhui Normal University, Wuhu, China; ^4^Wuhu Center for Disease Control and Prevention, Wuhu, China

**Keywords:** *Postn*, myotonic dystrophy type 1, microenvironment, myoblast, myogenesis

## Abstract

Myotonic dystrophy type 1 (DM1) is an inherited neuromuscular disease caused by expanded CTG repeats in the 3′ untranslated region (3′UTR) of the *DMPK* gene. The myogenesis process is defective in DM1, which is closely associated with progressive muscle weakness and wasting. Despite many proposed explanations for the myogenesis defects in DM1, the underlying mechanism and the involvement of the extracellular microenvironment remained unknown. Here, we constructed a DM1 myoblast cell model and reproduced the myogenesis defects. By RNA sequencing (RNA-seq), we discovered that periostin (*Postn*) was the most significantly upregulated gene in DM1 myogenesis compared with normal controls. This difference in *Postn* was confirmed by real-time quantitative PCR (RT-qPCR) and western blotting. Moreover, *Postn* was found to be significantly upregulated in skeletal muscle and myoblasts of DM1 patients. Next, we knocked down *Postn* using a short hairpin RNA (shRNA) in DM1 myoblast cells and found that the myogenesis defects in the DM1 group were successfully rescued, as evidenced by increases in the myotube area, the fusion index, and the expression of myogenesis regulatory genes. Similarly, *Postn* knockdown in normal myoblast cells enhanced myogenesis. As POSTN is a secreted protein, we treated the DM1 myoblast cells with a POSTN-neutralizing antibody and found that DM1 myogenesis defects were successfully rescued by POSTN neutralization. We also tested the myogenic ability of myoblasts in the skeletal muscle injury mouse model and found that *Postn* knockdown improved the myogenic ability of DM1 myoblasts. The activity of the TGF-β/Smad3 pathway was upregulated during DM1 myogenesis but repressed when inhibiting *Postn* with a *Postn* shRNA or a POSTN-neutralizing antibody, which suggested that the TGF-β/Smad3 pathway might mediate the function of *Postn* in DM1 myogenesis. These results suggest that *Postn* is a potential therapeutical target for the treatment of myogenesis defects in DM1.

## Introduction

Myotonic dystrophy type 1 (DM1) is an autosomal inherited neuromuscular disease caused by aberrant expanded (CTG) trinucleotide repeats in the 3′ untranslated region (3′UTR) of the *DMPK* gene. The copy number of CTG repeats is higher than 50 in DM1 patients but lower than 37 in healthy individuals. The individuals with 38–49 CTG repeats are considered to have premutations. The expanded CTG repeats in DM1 are transcribed, along with the *DMPK* gene, into mRNA containing expanded CUG repeats, referred to as “toxic RNA” (Udd and Krahe, 2012). This toxic RNA forms a hairpin-like secondary structure in cell nuclei, leading to MBNL1 sequestration (Miller et al., 2000) and CELF1 upregulation (Kuyumcu-Martinez et al., 2007; Kalsotra et al., 2010). Both MBNL1 and CELF1 are RNA-binding proteins and regulate alternative splicing of RNA. Thus, the dysregulation of *MBNL1* and *CELF1* leads to isoform switches of several important genes related to skeletal muscle function, including *CLCN1*, *BIN1, TNNT2*, *IR*, and *PKM*, which directly cause DM1 disease phenotypes (Philips et al., 1998; Savkur et al., 2001; Charlet et al., 2002; Mankodi et al., 2002; Ho et al., 2004; Fugier et al., 2011). Among these phenotypes, the myogenesis defect is a particularly serious problem in DM1 as it has been shown to be closely related to progressive muscle weakness and wasting (Kanadia et al., 2003; Ward et al., 2010).

Myogenesis is a complicated and precisely regulated process that produces myotubes of skeletal muscle. Many myogenic regulatory factors (MRFs) have been documented, including *MyoD*, *MyoG*, and *Mrf4* (Hernandez-Hernandez et al., 2017). Myogenesis consists of two stages, cell cycle withdrawal and myoblast fusion (Andre et al., 2018). In the initial stage, myoblast proliferation is required to generate sufficient cells for myoblast fusion. The proliferation process, however, should be terminated to enable the subsequent myogenesis process (Andres and Walsh, 1996). This cell cycle withdrawal is governed by *p21* (Halevy et al., 1995) and *Rb* (Zacksenhaus et al., 1996). Following cell cycle arrest, the myoblasts undergo cell fusion to generate multinucleated myotubes (Schnorrer and Dickson, 2004). Although the detailed mechanism remains elusive, many fusion-related regulators have been discovered, including *Myomaker* (Millay et al., 2013, 2014) and *Myomixer* (Bi et al., 2017). Many studies have proposed possible explanations for the defective myogenesis in DM1. *Celf1* is directly phosphorylated and regulated by *Akt* and *cyclin D3*/*cdk4*, which leads to *CCND1* upregulation and *p21* downregulation and causes impaired myogenesis in DM1 (Timchenko et al., 2001a; Salisbury et al., 2008). Consistently, recent studies have observed suppression of cell cycle withdrawal in DM1 or *Celf1*-overexpressing myoblasts (Furling et al., 2001; Peng et al., 2015), probably due to dysregulation of *cyclin D1* and *p21*. *DMPK*, a rho kinase, may be involved in the regulation of myosin light chain phosphorylation, and its isoform E has been shown to be crucial for normal muscle development (Jansen et al., 1996; Mulders et al., 2011). Although studies have shown that *DMPK* is dispensable for myoblast differentiation (Jansen et al., 1996), the *DMPK* dysregulation that occurs as a result of expanded CUG repeats suggests a potential role of this gene in myoblast differentiation. Moreover, MRFs including *MyoD* and *Six5* are altered in DM1 owing to the expanded CUG repeats and *DMPK* dysregulation, respectively (Inukai et al., 2000; Apponi et al., 2011). Although each of the above findings can partially explain the defective myogenesis in DM1, the underlying mechanism remains unclear.

Periostin (*Postn*) is a matricellular protein that consists of seven domains: a signal peptide, a cysteine-rich domain, a C-terminal region, and four FAS1 domains. *Postn* is well known as an important microenvironment component that favors tumor growth and metastasis. In ovarian cancer, *Postn* is upregulated by the TGF-β pathway and promotes migration and invasion (Yue et al., 2021). *Postn* is also a candidate prognostic marker in colorectal cancers (Oh et al., 2017) and promotes colorectal cancer progression through activating *YAP*/*TAZ* (Ma et al., 2020). In glioma, *Postn* promotes tumor growth, epithelial–mesenchymal transition (EMT), invasion, and resistance to antiangiogenic therapy by recruiting M2 macrophages and activating *STAT3* (Zhou et al., 2015; Park et al., 2016). *Postn* is targeted by *miR-876* and facilitates EMT and fibrosis of hepatocellular carcinoma (Chen et al., 2020). *Postn* also plays an important part in cancer stem cell maintenance by recruiting Wnt ligands to enhance Wnt signaling in cancer stem cells (Malanchi et al., 2011). In addition to its roles in cancer, *Postn* has been reported to regulate skeletal muscle regeneration; it is temporally expressed during skeletal muscle regeneration (Ozdemir et al., 2014), and *Postn* knockout improves muscle recovery and inhibits fibrosis after skeletal muscle injuries. Moreover, POSTN-neutralizing antibody treatment promotes recovery from skeletal muscle injuries in a mouse model (Hara et al., 2018). In a muscular dystrophy mouse model, *Postn* knockout was found to improve myogenesis and inhibit fibrosis by upregulating the TGF-β pathway (Lorts et al., 2012). Nevertheless, the function of *Postn* in regulating DM1 has remained unknown.

In this study, we used a DM1 mouse myoblast cell model to study myogenesis defects in DM1. *Postn* was found to be significantly upregulated both during the DM1 myoblast differentiation process and in skeletal muscles and myoblasts of DM1 patients. By downregulating *Postn* with short hairpin RNA (shRNA) or a neutralizing antibody, the myogenesis defects in DM1 were successfully rescued. Moreover, *Postn* knockdown in DM1 myoblasts improved the efficiencies of myogenesis and regeneration in a skeletal muscle injury mouse model. The TGF-β/Smad3 pathway that was enhanced in the DM1 myogenesis process was suppressed with *Postn* inhibitions, which might mediate the function of *Postn* in the myogenesis process of DM1 myoblasts. These results suggest that *Postn* is a potential therapeutical target for the treatment of DM1.

## Materials and Methods

### Cell Culture

C2C12 cells (RRID: CVCL_0188) were provided by the Stem Cell Bank, Chinese Academy of Sciences. C2C12 cells were cultured in high-glucose Dulbecco’s Modified Eagle Medium (DMEM, HyClone, Cat #SH30022.01) supplemented with 20% fetal bovine serum (Clark, Cat #FB15015), 50 U/mL penicillin (Biosharp, Cat #BL505A), and 50 μg/mL streptomycin (Biosharp, Cat #BL505A). *In vitro* myoblast differentiation was induced by switching the above medium to high-glucose DMEM (HyClone) supplemented with 2% horse serum (HyClone, Cat #SH30074.03), 50 U/mL penicillin (Biosharp), 50 μg/mL streptomycin (Biosharp), and 1 μM insulin (Beyotime, Cat#P3376-100IU) when cells were confluent. The *in vitro* myoblast differentiation process typically spanned 6 days. When neutralizing the secreted POSTN during myoblast differentiation, 1.5 μg/ml anti-POSTN antibody (Sino Biological, Cat #50450-RP02, RRID: AB_2891098) was added to the differentiation medium. 1.5 μg/ml IgG control antibody (Santa Cruz Biotechnology, Cat #sc-2025, RRID: AB_737182) was used as control. Both antibodies were added from day 0 to day 6 of *in vitro* myoblast differentiations.

### Construction of Plasmids and Cell Lines

The pcDNA-GFP-(CUG)_5_ (GFP-CUG5) and pcDNA-GFP-(CUG)_200_ (GFP-CUG200) plasmids were as described previously (Amack and Mahadevan, 2001). The pLL4.0 vector was previously developed by our laboratory (Shen et al., 2020). The pLL4.0 vector was constructed by replacing a CMV-EGFP cassette in the pLL3.7 vector with a PGK-puromycin cassette. Scrambled, shPostn, and shMbnl1 plasmids were generated by ligating the scrambled, *Postn*, and *Mbnl1* shRNA coding sequences into the pLL4.0 vector, respectively. The sequences of the scrambled, *Postn*, and *Mbnl1* shRNAs are listed in Supplementary Table 1.

Plasmids were transduced into cells using PolyJet (SignaGen, Cat #SL100688) according to the manufacturer’s instructions. Normal (C2C12 GFP-CUG5) and DM1 (C2C12 GFP-CUG200) myoblast cell models were produced by transfecting C2C12 cells with GFP-CUG5 and GFP-CUG200 plasmids, respectively, followed by G418 selection until stable. Control and *Postn* knockdown DM1 myoblast cell lines were produced by transfecting C2C12 GFP-CUG200 cells with the scrambled and shPostn plasmids, respectively, followed by puromycin selection until stable. Control and *Postn* knockdown normal myoblast cell lines were produced by transfecting C2C12-CUG5 cells with the scrambled and shPostn plasmids, respectively, followed by puromycin selection until stable. Control and *Mbnl1* knockdown myoblast cell lines were produced by transfecting C2C12 cells with the scrambled and shMbnl1 plasmids, respectively, followed by puromycin selection until stable.

### Total RNA Extraction and Real-Time Quantitative PCR

Total RNA was extracted using Total RNA Isolation Reagent (Biosharp, Cat #BS259A). Reverse transcription was performed using the FastKing RT Kit (Tiangen, Cat #KR118-02). Quantitative PCR was performed using the Powerup SYBR Master Mix (Applied Biosystems, Cat #A25778). These experiments were conducted according to the corresponding manufacturer’s manuals. *Gapdh* was used as a normalized control gene. The primer sequences used in real-time quantitative PCR (RT-qPCR) are listed in Supplementary Table 1.

### RNA Sequencing and Data Analysis

The library construction and sequencing steps of RNA sequencing (RNA-seq) were performed by Anhui Microanaly Genetech Co., Ltd. Raw data were subjected to adapter trimming and read filtering using the trim_galore software (Trim Galore, RRID: SCR_011847). The filtered data were aligned to the mouse genome (GRCm38) using Hisat2 (HISAT2, RRID: SCR_015530) and then analyzed with StringTie (RRID: SCR_016323) to generate readcount tables. Differentially expressed genes (DEGs) were determined by DESeq2 (RRID: SCR_015687) (Love et al., 2014) using ∣log2(fold change)∣ > 1 and adjusted *P*-value < 0.05 as the cutoffs. Gene ontology (GO) and Kyoto Encyclopedia of Genes and Genomes (KEGG) analyses were performed using the clusterProfiler package (clusterProfiler, RRID: SCR_016884) (Yu et al., 2012). RNA-seq data generated during this study are deposited at the Gene Expression Omnibus (GEO) database (GSE174119). The RNA-seq data of tibialis anterior (TA) muscles and myoblasts from healthy, and DM1 individuals were obtained from the GEO database using accession numbers GSE86356 and GSE158216, respectively.

### Protein Extraction and Western Blotting

Intracellular protein samples were extracted using Cell Lysis Buffer (Beyotime, Cat #P0013) supplemented with EASYpack Protease Inhibitors (Roche, Cat #5892970001). Protein concentrations were measured with a BCA protein assay kit (Biosharp, Cat # BL521A) and then adjusted to be the same. Supernatant protein samples were obtained by collecting the culture medium of the corresponding cells. For normalization, the volumes of the cell culture medium were initially the same when culturing cells and the loading volumes of the culture medium were normalized to their corresponding cell numbers when doing gel electrophoresis. Samples were subjected to sodium dodecyl sulfate polyacrylamide gel electrophoresis and the proteins were transferred onto PVDF membranes. The membranes were then blocked and incubated with primary antibodies overnight at 4°C. On the next day, the membranes were incubated with horseradish peroxidase (HRP)-conjugated secondary antibodies and reacted with chemiluminescent substrates (Biosharp, Cat #BL520A). Images were taken with a Tanon 5200 Imaging System. The antibodies and dilutions were as follows: anti-POSTN pAb (1:1,000, Sino Biological, Cat #50450-RP02, RRID: AB_2891098), anti-SMAD3 (1:2,000, Santa Cruz, Cat #sc-101154, RRID: AB_1129525), anti-p-SMAD3 (1:2,000, Santa Cruz, Cat #sc-517575, RRID: AB_2892229), anti-MBNL1 mAb (1:2,000, Novus, Cat #NB110-37256, RRID: AB_792678), anti-GAPDH pAb (1:2,000, Biosharp, Cat #BL006B, RRID: AB_2890028), goat anti-mouse HRP antibody (1:2,000, Biosharp, Cat #BL001A, RRID: AB_2827665), and donkey anti-rabbit HRP antibody (1:2,000, Invitrogen, Cat #31458, RRID: AB_228213). The intensities of the western blot gel bands were measured using ImageJ (RRID: SCR_003070).

### Immunostaining

Samples (cells and slides) were fixed with 4% paraformaldehyde at room temperature. After that, the samples were blocked with the blocking solution (10% normal goat serum and 0.1% Triton X-100 in PBS). The samples were then incubated with primary antibodies that were diluted in the blocking solution at 4°C overnight. On the next day, the samples were incubated with fluorescence conjugated secondary antibodies and DAPI. The antibodies and dilutions were as follows: anti-myosin heavy chain (MHC) mAb (1:10, DHSB, Catalog No. AB_2147781, and RRID: AB_2147781) and goat anti-mouse Alex Fluor Plus 555-conjugated IgG (1:500, Invitrogen, Catalog No. A32727, and RRID: AB_2633276). All images were obtained with a Leica DMi8 fluorescence microscope and analyzed with ImageJ (RRID: SCR_003070). Fusion index equaled to the ratio of nuclei number in the cells with at least two nuclei vs. total nuclei number. Myotube area equaled to the ratio of the MHC fluorescence positive area vs. the whole area in the immunostaining images.

### Mice and Skeletal Muscle Injury Models

All mouse-related experiments were performed according to the protocols approved by the Institutional Animal Care and Use Committee of Anhui Normal University. Eight-week old male Swiss mice were anesthetized and injected with 25 μl of 10 μM cardiotoxin (CTX, Sigma, Cat #217503) into TA muscles to produce skeletal muscle injury models. On the next day, the CTX injected TA muscles were injected with scramble control and *Postn* knockdown DM1 myoblast cells (5 × 10^4^ cells per TA muscle), respectively, to test their myogenic abilities *in vivo*. PBS was used as a sham control. The TA muscles were harvested 14 days after the cell injections and subjected to cryosectioning using OCT (Sakura, Cat #4583). The slices of TA muscles were then stained with hematoxylin & eosin (H&E, Biosharp, Cat #BL700B) and immunostained against MHC to determine muscle regeneration efficiencies after injury.

### Statistical Analysis

All experiments were performed at least three times. Shapiro–Wilk test was used for data normality test. Student’s *t*-test was used for two-group comparisons, and one-way analysis of variance (ANOVA) followed by *post hoc* Tukey tests was used for comparisons of three or more groups. An asterisk is used to label significant differences (*P* < 0.05) in the figures. All data are presented as mean ± SD.

## Results

### Myogenesis Was Significantly Impaired in the DM1 Myoblast Cell Model

**FIGURE 1:**
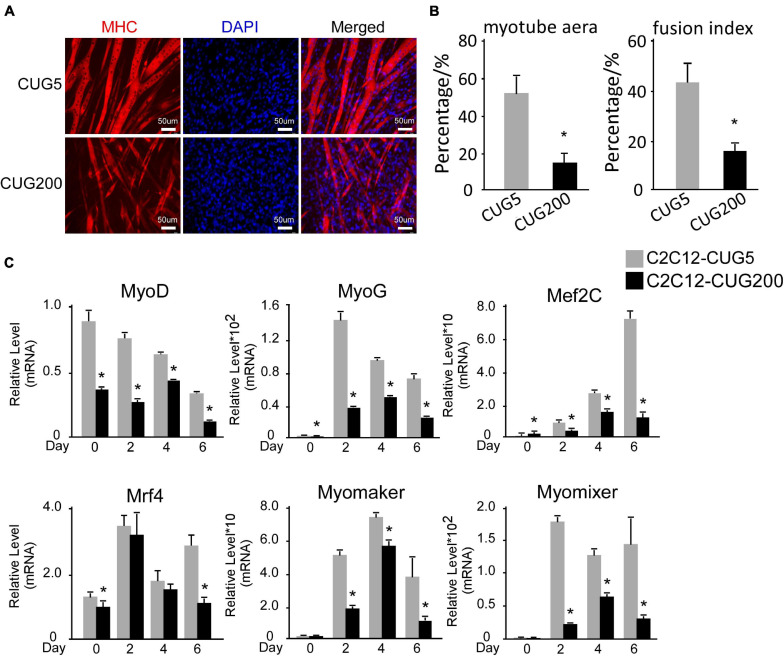
Myogenesis was significantly impaired in the myotonic dystrophy type 1 (DM1) myoblast cell model. **(A)** Myotube formation by myoblast cells of both normal control and DM1 groups, detected by immunostaining against myosin heavy chain (MHC) at differentiation day 6. **(B)** Myotube area and fusion index in both the normal control and DM1 groups quantified with ImageJ software. **(C)** Expression levels of myogenic regulatory factors (MRFs; *MyoD*, *MyoG*, *Mef2C*, and *Mrf4*) and fusion markers (*Myomaker* and *Myomixer*) in normal control, and DM1 groups, measured by real-time quantitative PCR (RT-qPCR). All expression levels were normalized to the values of the normal control group at day 0. CUG5, normal control myoblast cells (C2C12 GFP-CUG5); CUG200, DM1 myoblast cells (C2C12 GFP-CUG200); *P < 0.05.

The myogenesis process is severely impaired in DM1 according to most studies (Amack et al., 2002; Timchenko et al., 2004; Kuyumcu-Martinez et al., 2007; Peng et al., 2015), although several groups have reported no significant change in myogenic abilities in myoblasts derived from some DM1 patients (Jacobs et al., 1990; Loro et al., 2010). Therefore, we first compared the myogenic abilities of DM1 and normal murine myoblast cell models. To construct DM1 and normal myoblast cell models, we stably transfected murine myoblast C2C12 cells with the GFP-CUG5 and GFP-CUG200 plasmids, respectively. Normal and DM1 myoblasts were subjected to *in vitro* myoblast differentiation. At differentiation day 6, the DM1 group displayed markedly less myotube formation compared with the normal control, as visualized by immunostaining against MHC (Figure 1A). The myotube area was 51.69% ± 9.51% in the normal group but 14.25% ± 5.24% in the DM1 group; and the fusion index was 43.20% ± 7.34% in the normal group but 15.72% ± 2.77% in the DM1 group (Figure 1B). Through RT-qPCR, we found that MRFs (*MyoD*, *MyoG*, *Mef2C*, and *Mrf4*) were significantly inhibited in the DM1 group during *in vitro* myoblast differentiation. Moreover, the essential myoblast fusion markers *Myomaker* and *Myomixer* were also downregulated (Figure 1C). These results confirmed that myoblast differentiation and fusion were both impaired in DM1 myoblasts.

### Periostin Might Mediate Aberrant Myogenesis in DM1 Myoblasts

**FIGURE 2:**
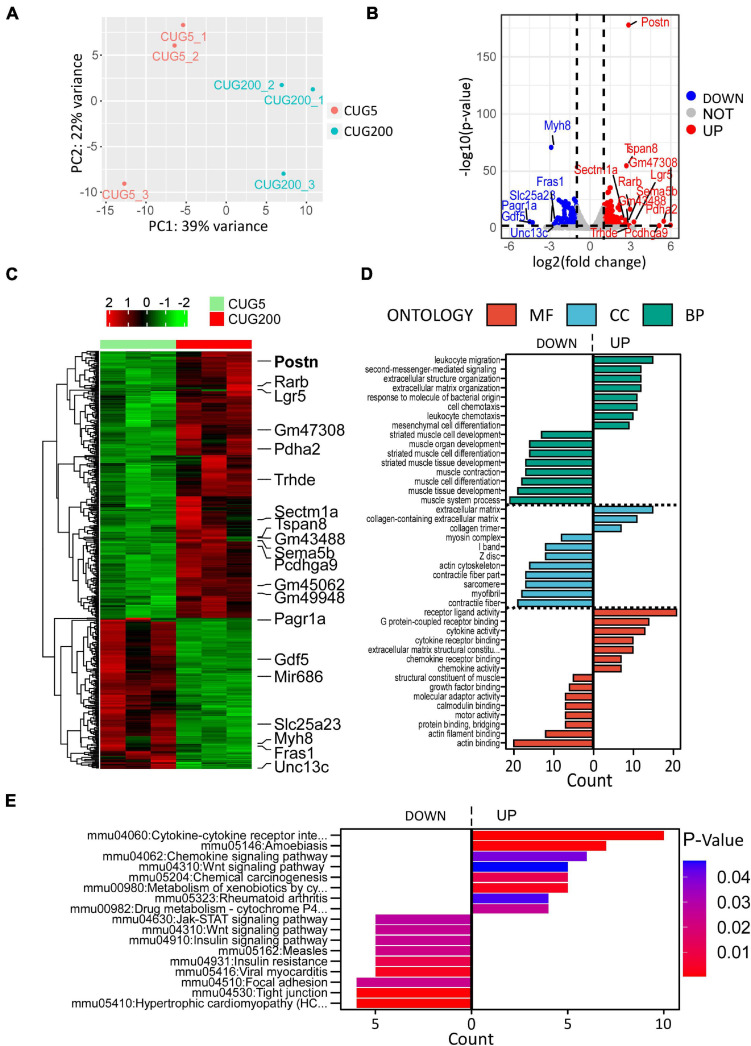
Periostin (*Postn*) was the top significantly altered gene in DM1 myoblast differentiation compared with the normal control. **(A)** Principal components analysis (PCA) of RNA sequencing (RNA-seq) data from normal and DM1 groups. Three biological replicates are included for each group. **(B)** Volcano plot showing the differentially expressed genes (DEGs). *Postn* was the top significantly altered gene. **(C)** Heatmap showing relative levels of all DEGs in both groups. **(D)** Gene ontology (GO) analysis of the DEGs. **(E)** Kyoto Encyclopedia of Genes and Genomes (KEGG) analysis of the DEGs. CUG5, normal control myoblast cells (C2C12 GFP-CUG5); CUG200, DM1 myoblast cells (C2C12 GFP-CUG200); MF, molecular function; CC, cellular component; and BP, biological process.

Although many studies have proposed possible explanations for the myogenesis defects in DM1, the underlying mechanism, especially the involvement of the microenvironment, has remained unclear. To investigate this mechanism, we performed RNA-seq on total RNA samples of normal and DM1 myoblasts at differentiation day 4, when myotubes started to form during *in vitro* myoblast differentiation. Principal components analysis (PCA) indicated that the gene expression patterns between normal and DM1 groups were different (Figure 2A). Next, we analyzed DEGs of the two groups using DESeq2, with ∣log2(fold change)∣ > 1 and adjusted *P*-value < 0.05 as the cutoffs for DEG determination. There were 279 upregulated and 158 downregulated genes in the DM1 vs. the normal group (Figure 2B). As shown in the heatmap of relative levels of all DEGs in Figure 2C, *Postn* was markedly upregulated in the DM1 group. Table 1 shows the top 20 level-changed genes in the DM1 group. *Pdha2*, *Pcdhga9*, *Lgr5*, *Rarb*, *Trhde*, *Postn*, *Sema5b*, *Tspan8*, and *Sectm1a* were significantly upregulated, while *miR-686*, *Pagr1a*, *Gdf5*, *Myh8*, *Slc25a23*, *Unc13c*, and *Fras1* were significantly downregulated. *Postn* was the most significantly altered gene, with log2(fold change) = 2.86 and adjusted *P*-value = 1.79E−178. We then performed GO and KEGG analyses on all DEGs. The GO results showed that all striated muscle-related biological processes (BP), cellular components (CC), and molecular functions (MF) were inhibited (Figure 2D). The KEGG results showed that striated muscle-related pathways (Jak-STAT signaling, insulin signaling, and insulin resistance) were significantly repressed. Surprisingly, some components of the Wnt signaling pathway were upregulated but some other components were downregulated (Figure 2E). In summary, *Postn* was the most significantly upregulated gene in the DM1 group, implying that *Postn* might be associated with DM1 pathogenesis.

**TABLE 1:** Top altered genes in RNA sequencing (RNA-seq).

Gene	log2(fold change)	Adjusted *P*-value	Direction of change
*Gm45062*	11.03	2.55E−02	Upregulated
*Gm49948*	8.25	1.93E−06	Upregulated
*Gm47308*	5.98	2.02E−03	Upregulated
*Pdha2*	5.48	6.79E−07	Upregulated
*Pcdhga9*	5.14	5.18E−03	Upregulated
*Lgr5*	3.26	2.93E−06	Upregulated
*Rarb*	2.97	1.93E−17	Upregulated
*Trhde*	2.94	6.42E−03	Upregulated
*Postn*	2.86	1.79E−178	Upregulated
*Sema5b*	2.71	4.62E−06	Upregulated
*Tspan8*	2.70	2.18E−55	Upregulated
*Sectm1a*	2.64	7.53E−08	Upregulated
*Gm43488*	2.62	3.34E−04	Upregulated
*miR-686*	–7.41	8.74E−08	Downregulated
*Pagr1a*	–4.49	1.99E−06	Downregulated
*Gdf5*	–4.29	9.99E−06	Downregulated
*Myh8*	–2.90	1.97E−71	Downregulated
*Slc25a23*	–2.70	3.39E−04	Downregulated
*Unc13c*	–2.58	3.32E−02	Downregulated
*Fras1*	–2.54	8.09E−07	Downregulated

**FIGURE 3:**
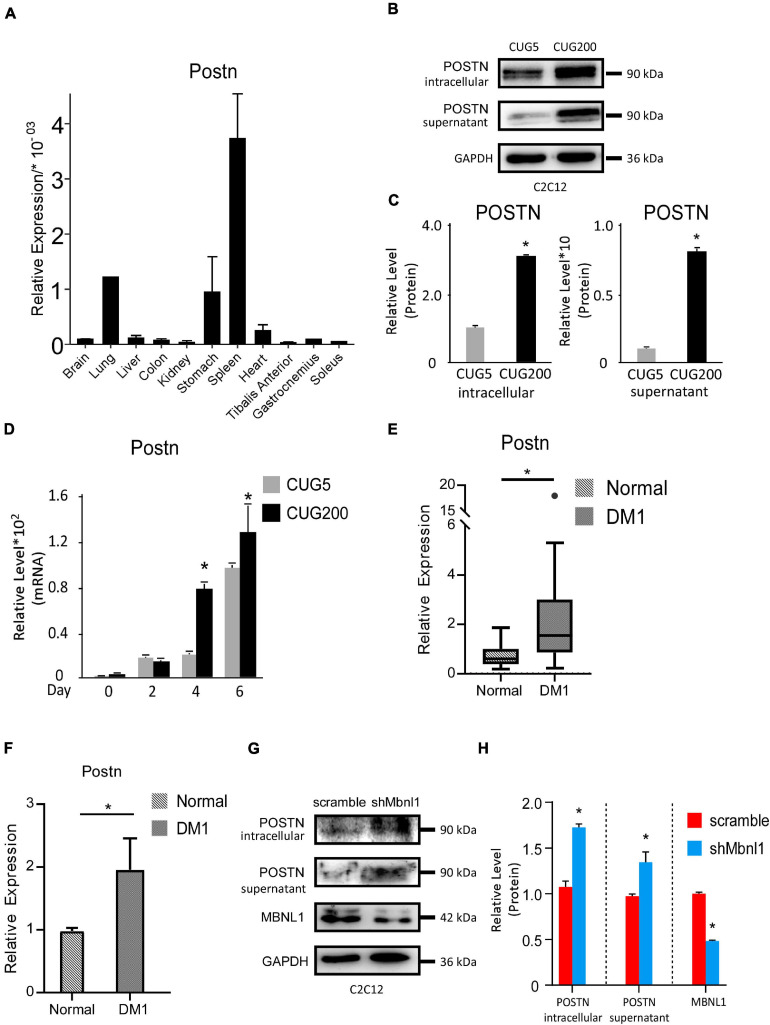
Periostin was upregulated in DM1 myogenesis and in skeletal muscle of DM1 patients. **(A)** Expression levels of *Postn* in different tissues of normal adult mice, measured by RT-qPCR. **(B)** Intracellular and supernatant POSTN expression levels in normal and DM1 myoblast cells, detected by western blotting. **(C)** Gel band intensities from panel **(B)** quantified using ImageJ software. Values were normalized to GAPDH. **(D)** Expression levels of *Postn* during *in vitro* myoblast differentiation, measured by RT-qPCR. All expression levels were normalized to the values of the normal control at day 0. **(E)** Expression levels of *Postn* in tibialis anterior (TA) muscle from healthy individuals (*n* = 10) and DM1 patients (*n* = 40). **(F)** Expression levels of *Postn* in myoblasts from healthy individuals (*n* = 3) and DM1 patients (*n* = 3). **(G)** MBNL1, intracellular and supernatant POSTN expression levels in scramble control and *Mbnl1* knockdown myoblast cells, detected by western blotting. **(H)** Gel band intensities from panel **(G)** quantified using ImageJ software. Values were normalized to GAPDH. CUG5, normal control myoblast cells (C2C12 GFP-CUG5); CUG200, DM1 myoblast cells (C2C12 GFP-CUG200); scramble, scramble control myoblast cells; and shMbnl1, Mbnl1 knockdown myoblast cells; *P < 0.05.

We then studied the expression levels of *Postn* in various tissues of normal adult mice. *Postn* was highly expressed in spleen, lung, and stomach but showed relatively low expression in skeletal muscle (TA, gastrocnemius, and soleus) (Figure 3A). To verify the changes in *Postn* levels observed by RNA-seq, we first performed western blotting against POSTN at differentiation day 4 for normal and DM1 myoblasts. As POSTN is a secreted protein, we detected both intracellular and supernatant POSTN levels. Both intracellular and supernatant POSTN were upregulated in DM1 (Figures 3B,C). Next, we checked the expression pattern of *Postn* during myoblast differentiation. *Postn* was significantly upregulated from days 4 to 6 of myoblast differentiation in DM1 compared with the normal group (Figure 3D). We then investigated whether *POSTN* was also upregulated in the skeletal muscle of DM1 patients. We analyzed an RNA-seq dataset for TA muscle of healthy individuals (*n* = 10) and DM1 patients (*n* = 40) from the DMseq Deep Sequencing Data Repository^1^ and found a significant upregulation of *POSTN* in the DM1 group (Figure 3E). Moreover, by analyzing the RNA-seq data of myoblasts from healthy and DM1 individuals, we also observed a significant upregulation of *POSTN* in the myoblasts of DM1 patients (Figure 3F). Next, we studied if the *Postn* upregulation correlated with the *Mbnl1* downregulation in DM1. By western blotting, we found that the intracellular and secreted POSTN were both significantly upregulated with *Mbnl1* knockdown in C2C12 cells (Figures 3G,H). These results suggest a correlation between DM1 pathogenesis and *Postn* upregulation.

### Downregulation of *Postn* Using shRNA Rescued Myogenesis Defects in DM1

**FIGURE 4:**
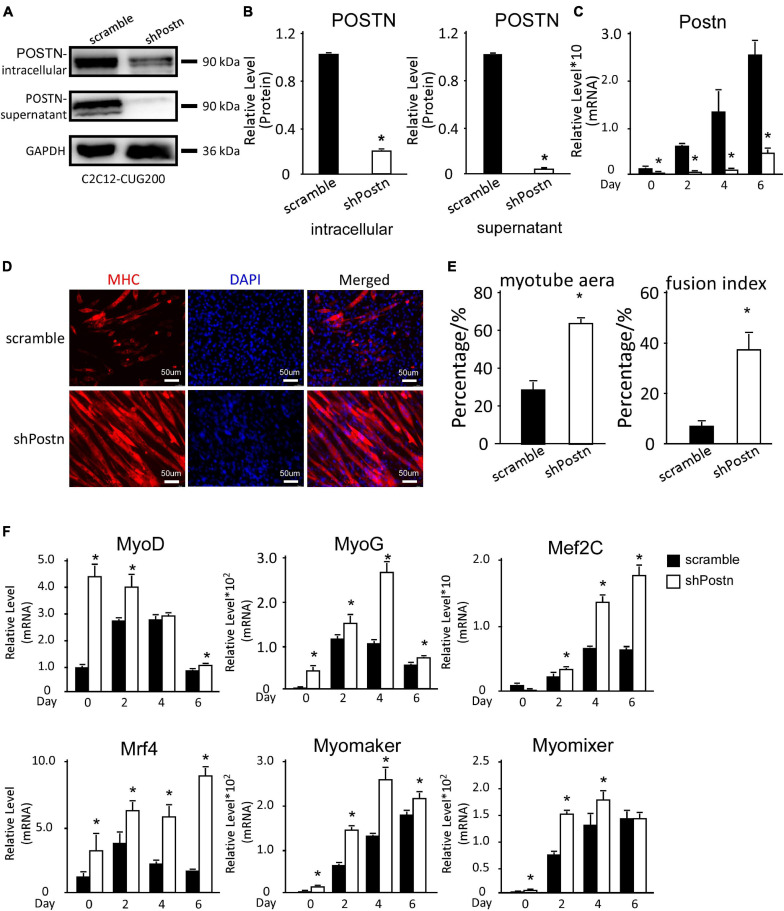
Periostin knockdown rescued the myogenesis defects in DM1 myoblast cells. **(A)** *Postn* knockdown in DM1 myoblast cells (C2C12 GFP-CUG200) verified by western blotting. **(B)** Gel band intensities from panel **(A)** quantified using ImageJ software. Values were normalized to GAPDH. **(C)** Expression levels of *Postn* during differentiation in both scrambled control and *Postn*-knockdown groups, measured by RT-qPCR. **(D)** Myotube formation in both scrambled control and *Postn*-knockdown groups, detected by immunostaining against MHC at differentiation day 6. **(E)** Myotube area and fusion index in both groups quantified using ImageJ software. **(F)** Expression levels of MRFs (*MyoD*, *MyoG*, *Mef2C*, and *Mrf4*) and fusion markers (*Myomaker* and *Myomixer*) in both groups, measured by RT-qPCR. All expression levels were normalized to the values of the scrambled control at day 0. Scramble, scrambled control DM1 myoblast cells; shPostn, *Postn*-knockdown DM1 myoblast cells; *P < 0.05.

As *Postn* was aberrantly upregulated in DM1 myoblast differentiation, we investigated whether downregulation of *Postn* could rescue the myogenesis defect in DM1. We constructed scrambled control and *Postn*-knockdown DM1 myoblast cell lines by stably transfecting C2C12 GFP-CUG200 cells with the scrambled and shPostn plasmids, respectively. Western blots showed that both intracellular and supernatant POSTN were significantly downregulated in *Postn*-knockdown DM1 myoblast cells (Figures 4A,B). The *Postn* knockdown and control DM1 myoblast cells were subjected to *in vitro* myoblast differentiation. RT-qPCR showed that *Postn* was significantly downregulated throughout the differentiation process (Figure 4C). Immunostaining against MHC showed that *Postn* knockdown robustly improved myotube production (Figure 4D). The myotube area was 61.32% ± 2.58% in the *Postn* knockdown group but 27.53% ± 4.13% in the scrambled control group; and the fusion index was 41.96% ± 7.38% in the *Postn* knockdown group but 7.64% ± 2.32% in the scrambled control group (Figure 4E). Consistently, MRFs (*MyoD*, *MyoG*, *Mef2C*, and *Mrf4*) were all markedly upregulated, and fusion markers (*Myomaker* and *Myomixer*) were also boosted (Figure 4F).

**FIGURE 5:**
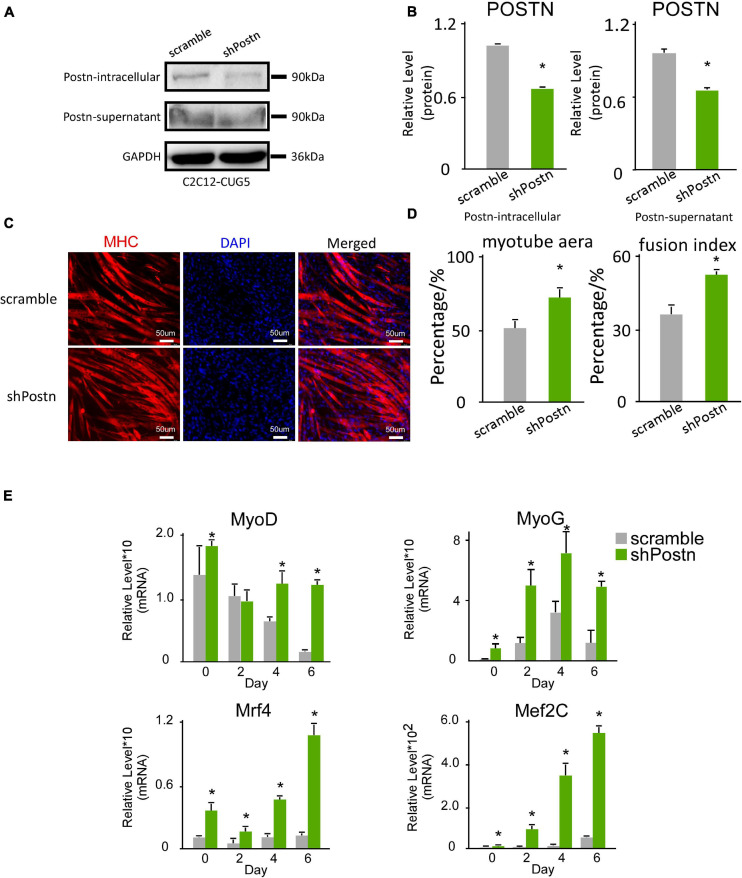
Periostin knockdown promoted myogenesis in normal myoblast cells. **(A)** *Postn* knockdown in normal myoblast cells (C2C12 GFP-CUG5), verified by western blots. **(B)** Gel band intensities from panel **(A)** quantified with ImageJ software. Values were normalized to GAPDH. **(C)** Myotube formation in both scrambled control and *Postn*-knockdown groups, detected by immunostaining against MHC at differentiation day 6. **(D)** Myotube area and fusion index in both groups quantified with ImageJ software. **(E)** Expression levels of MRFs (*MyoD*, *MyoG*, *Mef2C*, and *Mrf4*) in both groups, measured by RT-qPCR. All expression levels were normalized to the values of the scramble control at day 0. Scramble, scramble control normal myoblast cells; shPostn, *Postn* knockdown normal myoblast cells; *P < 0.05.

We also determined the effect of *Postn* inhibition on normal myoblast differentiation. *Postn*-knockdown and control normal myoblast cell lines were produced by stably transfecting C2C12 GFP-CUG5 cells with the shPostn and scrambled plasmids, respectively. The knockdown efficiency was verified by western blotting (Figures 5A,B). Next, we performed *in vitro* myoblast differentiation on these two cell lines. At differentiation day 6, we found that myotube formation in normal myoblast cells was enhanced by *Postn* knockdown, as indicated by immunostaining against MHC (Figure 5C). Myotube area and fusion index were both increased with *Postn* knockdown (Figure 5D), and the expression levels of MRFs (*MyoD*, *MyoG*, *Mef2C*, and *Mrf4*) were significantly elevated (Figure 5E). Taken together, besides rescuing myogenesis defect in DM1 myoblast cells, *Postn* inhibition in normal myoblast promotes the myogenesis process in normal myoblasts.

### Neutralizing Antibody Treatment Against POSTN Also Rescued Myogenesis Defects in DM1

**FIGURE 6:**
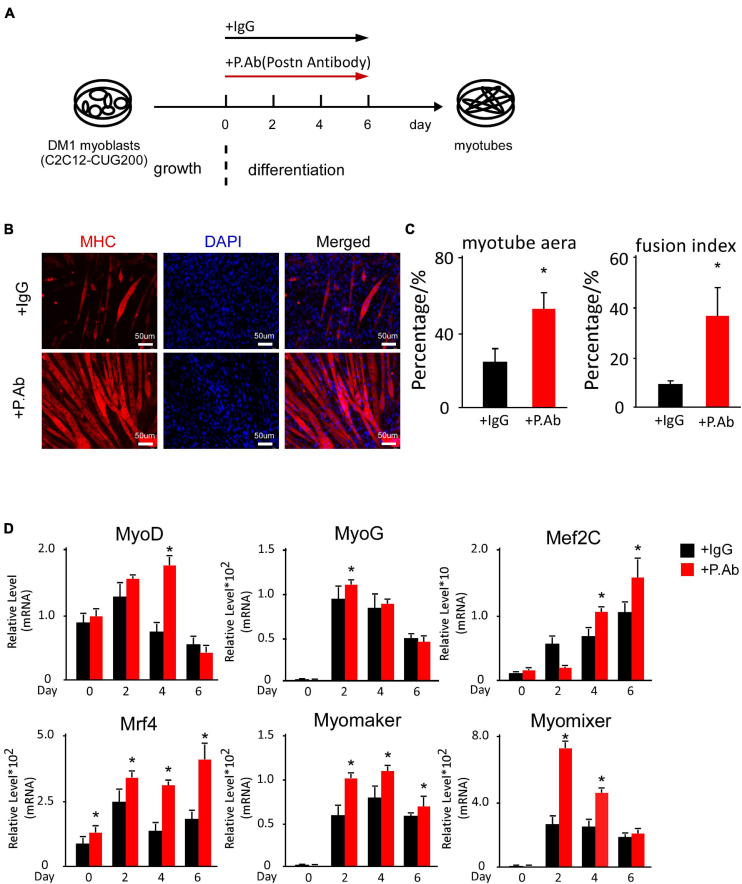
POSTN-neutralizing antibody treatment rescued myogenesis defects in DM1 myoblast cells. **(A)** Schematic diagram of the myoblast differentiation processes of DM1 myoblast cells treated with control IgG and POSTN-neutralizing antibody. **(B)** Myotube formation in both control IgG and POSTN-neutralizing antibody groups, detected by immunostaining against MHC at differentiation day 6. **(C)** Myotube area and fusion index in both groups quantified with ImageJ software. **(D)** Expression levels of MRFs (*MyoD*, *MyoG*, *Mef2C*, and *Mrf4*) and fusion markers (*Myomaker* and *Myomixer*) in both groups, measured by RT-qPCR. All expression levels were normalized to the values of the control IgG group at day 0. IgG, control IgG antibody; P.Ab, POSTN neutralizing antibody; *P < 0.05.

As *Postn* shRNA successfully rescued myogenesis defects in DM1, and POSTN is a secreted protein, we considered whether neutralizing excess extracellular POSTN could also rescue myogenesis defects in DM1. We performed *in vitro* myoblast differentiation on DM1 myoblast cells and treated them with a neutralizing antibody against POSTN and a control IgG, respectively (Figure 6A). Immunostaining against MHC at differentiation day 6 showed that myogenesis was improved with POSTN antibody treatment (Figure 6B). The myotube area was 56.27% ± 9.08% in the POSTN antibody group but 25.67% ± 7.74% in the control group; and the fusion index was 41.74% ± 12.92% in the POSTN antibody group but 10.22% ± 1.79% in the control group (Figure 6C). The RT-qPCR results showed that MRFs and fusion markers were upregulated with POSTN antibody treatment (Figure 6D). In conclusion, neutralizing excessive POSTN in the DM1 myoblast extracellular microenvironment could rescue myogenesis defects in DM1.

### Periostin Knockdown Improved the Myogenic Ability of DM1 Myoblasts *in vivo*

**FIGURE 7:**
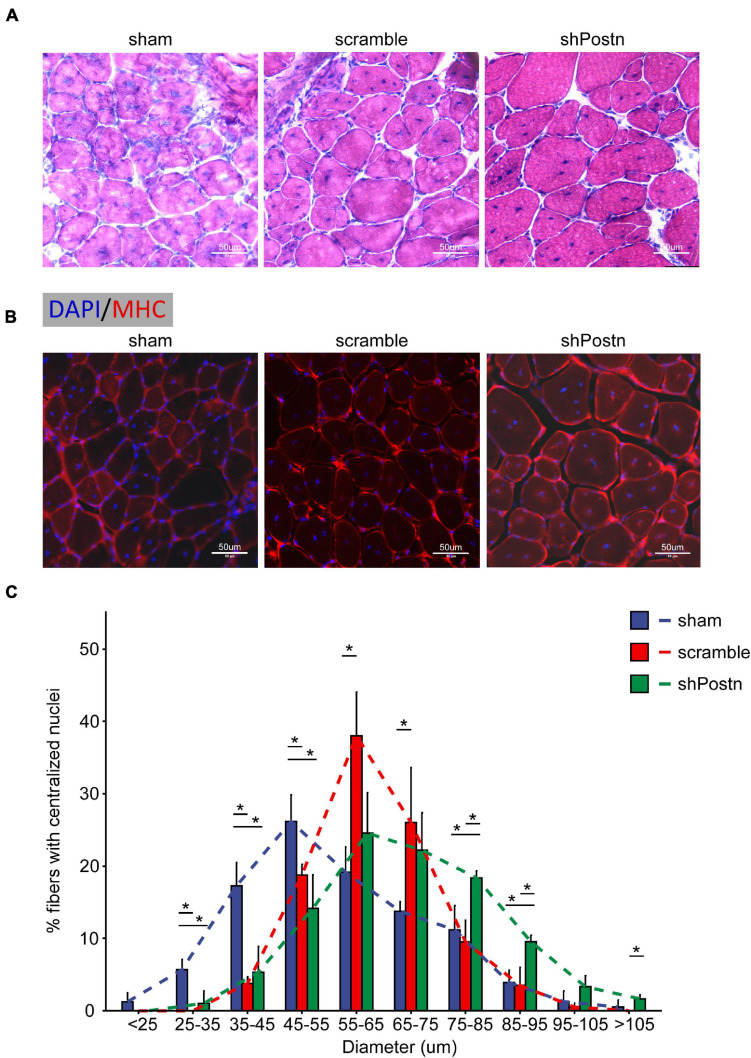
Knockdown improved the myogenic ability of DM1 myoblasts *in vivo*. **(A)** H&E staining of cross-sections of the TA muscles from the sham, scramble, and Postn knockdown groups. **(B)** Immunostaining against MHC of cross-sections of the TA muscles from the sham, scramble, and Postn knockdown groups. **(C)** The diameters of myofibers of the TA muscles from the sham, scramble control, and Postn knockdown groups. *S*ham, PBS; scramble, scramble control DM1 myoblasts; and shPostn, Postn knockdown DM1 myoblasts; *P < 0.05.

To determine whether *Postn* inhibition affected the myogenic ability of DM1 myoblasts *in vivo*, we injected scramble control and *Postn* knockdown DM1 myoblasts into the TA muscles that were treated with CTX to induce skeletal muscle injuries as described previously (Lee et al., 2015). The TA muscles were harvested 2 weeks after the cell injections. By HE staining and immunostaining against MHC, we found that the *Postn* knockdown DM1 myoblasts group displayed a better skeletal muscle morphology than the scramble control group, though the scramble control group also showed slight advantages over the sham control group (Figures 7A,B). The distributions of myotube size, on the whole, were the largest in the *Postn* knockdown group, the middle in the scramble control group, and the smallest in the sham group (Figure 7C). These results suggested that *Postn* knockdown improved the myogenic ability of DM1 myoblasts, which contributed to skeletal muscle regeneration *in vivo*.

### Periostin Regulated Myogenesis Likely Through the TGF-β/Smad3 Pathway in DM1 Myoblasts

**FIGURE 8:**
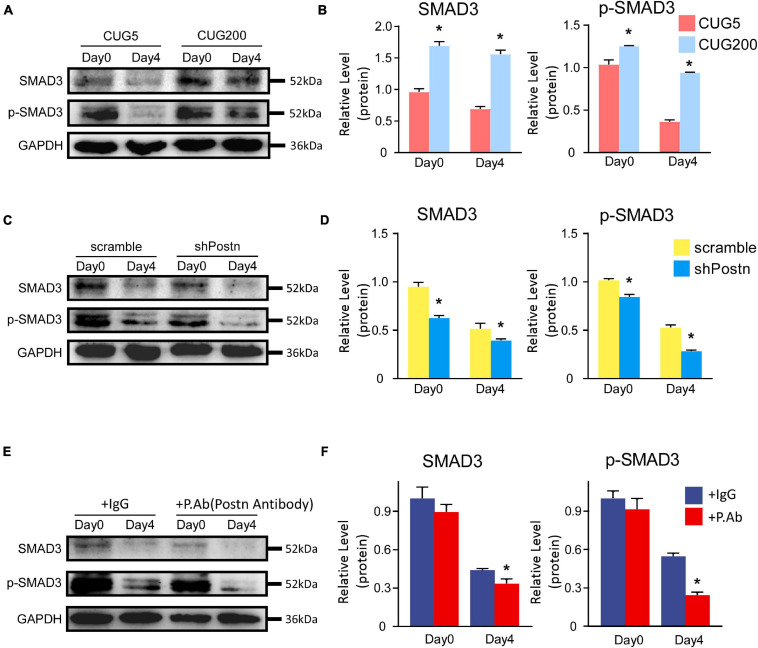
*Postn* regulated myogenesis likely through TGF-b/Smad3 pathway in DM1 myoblasts. **(A)** The levels of SMAD3 and p-SMAD3 in normal and DM1 myoblasts at differentiation day 0 and day 4 were determined by western blots. **(B)** Gel band intensities from **(A)** quantified with ImageJ software. GAPDH served as an internal control. All expression levels were normalized to the normal myoblasts at day 0. **(C)** The levels of SMAD3 and p-SMAD3 in scramble control and *Postn* knockdown DM1 myoblasts at differentiation day 0 and day 4 were determined by western blots. **(D)** Gel band intensities from **(C)** quantified with ImageJ software. **(E)** The levels of SMAD3 and p-SMAD3 in the control IgG antibody and POSTN neutralizing antibody treated DM1 myoblasts at differentiation day 0 and day 4 were determined by western blots. **(F)** Gel band intensities from **(E)** quantified with ImageJ software. GAPDH served as an internal control. All expression levels were normalized to the scramble control DM1 myoblasts at day 0. CUG5, normal control myoblast cells (C2C12 GFP-CUG5); CUG200, DM1 myoblast cells (C2C12 GFP-CUG200); scramble, scrambled control DM1 myoblast cells; shPostn, *Postn* knockdown DM1 myoblast cells; IgG, control IgG antibody; P.Ab, POSTN neutralizing antibody; *P < 0.05.

Periostin expression was reported to be controlled by the TGF-β/Smad pathway, which also, in turn, regulated the TGF-β/Smad pathway (Blanchard et al., 2008; Lorts et al., 2012; Noguchi et al., 2016; Mitamura et al., 2018; Yue et al., 2021). TGF-β inhibits the myogenesis process through Smad3 rather than Smad2 (Liu et al., 2001). Thus, we here investigated whether *Postn* regulated myogenesis through the TGF-β/Smad3 pathway in DM1 myoblasts. Compared to normal myoblasts, both p-SMAD3 and SMAD3 were upregulated in DM1 myoblasts at differentiation day 0 and day 4 (Figures 8A,B). When inhibiting *Postn* using shRNA, both p-SMAD3 and SMAD3 were downregulated in DM1 myoblasts (Figures 8C,D). Similarly, both p-SMAD3 and SMAD3 were downregulated when DM1 myoblasts were treated with a POSTN-neutralizing antibody during myoblast differentiation (Figures 8E,F). These results suggested that *Postn* might regulate the myogenesis process in DM1 myoblasts through the TGF-β/Smad3 pathway.

## Discussion

In this study, we discovered that *Postn* was aberrantly upregulated during the myogenesis process of DM1 myoblast cells, particularly from *in vitro* differentiation day 4, when myotubes started to form as a result of myoblast fusion. Next, we downregulated *Postn* in DM1 myoblast cells using both shRNA and a neutralizing antibody and found that the inhibition of *Postn* significantly rescued myogenesis defects in DM1. Consistently, inhibiting *Postn* also improved the myogenic ability of DM1 myoblast cells in the skeletal muscle injury mouse model. The TGF-β/Smad3 pathway might mediate the function of *Postn* in the myogenesis process of DM1 myoblast cells. Moreover, we tested whether *Postn* downregulation also affected the myogenesis process of normal myoblast cells. Knockdown of *Postn* in normal myoblast cells significantly facilitated the myogenesis process. Taken together, these results show that *Postn*, which encodes an extracellular protein, mediates defective myogenesis in DM1, which contributes to our understanding of the DM1 pathogenic mechanism. Targeting extracellular *Postn* is a potential approach for the therapy of myogenesis defects in DM1, with advantages of delivery convenience compared with classical intracellular therapeutic strategies.

To study myogenesis defects in DM1, we employed a widely used DM1 mouse myoblast cell model, produced by stable transfection with a plasmid containing 200 copies of CTG repeats at the 3′UTR of the *GFP* gene. The control cell model was constructed with a plasmid containing five copies of CTG repeats at the 3′UTR of the *GFP* gene. In previous work, we validated the pathological features of DM1 in this DM1 myoblast model, including ribonuclear foci, aberrant alternative splicing, and defective myogenesis (Shen et al., 2020). Other studies have also suggested using this cell model to investigate myogenesis defects in DM1 (Timchenko et al., 2001b; Peng et al., 2015). Consistently, we observed similar DM1 myogenesis defects to those reported by prior studies.

To discover the DEGs during the DM1 myogenesis process, we performed RNA-seq on RNA samples from both the normal and DM1 groups. There were 279 upregulated and 158 downregulated genes in the DM1 group. Table 1 showed the top 20 level-changed genes in the DM1 group. To our best knowledge, none of these genes were reported to function in DM1 before. Four genes (*Lgr5*, *Gdf5*, *Myh8*, and *Unc13c*) were known to regulate skeletal muscle myogenesis and homeostasis: *Lgr5* is a marker for a group of activated satellite cells for muscle regeneration (Leung et al., 2020); *Gdf5* was found to promote myogenesis process in sciatic denervation mouse model (Traore et al., 2019); *Myh8*, encoding embryonic and neonatal type MHCs, are transient elevated following muscle injury (Schiaffino et al., 2015; Yoshimoto et al., 2020); *Unc13c* facilitates myogenesis process, whose expression is repressed by TNF-α (Meyer et al., 2015). Particularly, there were four genes (*Gm45062*, *Gm49948*, *Gm47308*, and Gm *43488*) upregulated in DM1, whose official gene symbols, however, had not been assigned yet. These top-altered genes deserved further investigations in the future, as their functions were mostly unclear in skeletal muscle and DM1. GO analysis showed that skeletal muscle-related processes and structures were repressed in DM1. These results confirmed the myogenesis defects in DM1 myoblast cells. Moreover, we found that *Postn* was the most significantly altered gene in the DM1 group, with log2(fold change) = 2.86 and adjusted *P*-value = 1.79E−178. The upregulation of DM1 was confirmed by western blots and RT-qPCR. Through analyzing the datasets from the GEO database, we also discovered that the expression of *Postn* was enhanced in skeletal muscle and myoblasts of DM1 patients. MBNL1 is sequestered by the toxic RNA in DM1, which results in the downregulation of active MBNL1 in cells. We here found that inhibiting *Mbnl1* using shRNA significantly upregulated the intracellular and secreted POSTN, which suggested a correlation between *Mbnl1* and *Postn*. This finding was consistent with the RNA-seq data from a recent study that showed upregulations of *Postn* in DM1 mice models (HSALR20b and Mbnl3/4KO mice) (Tanner et al., 2021). These results imply a potential regulatory role of *Postn* in DM1 pathogenesis. Previous studies have indicated that *Postn* could serve as serum biomarkers for many diseases, including cancer (Dong et al., 2018a,b), rhinosinusitis (Ninomiya et al., 2018), and asthma (Hachim et al., 2020). Based on the upregulations of *Postn* in the DM1 myoblast cell model and the skeletal muscle and myoblasts from DM1 patients, we thought *Postn* might be used as a biomarker for DM1, which, however, needed further verifications of the expressions of *Postn* in the serum of DM1 patients.

We then investigated whether downregulating *Postn* in DM1 myoblasts could rescue myogenesis defects. Knockdown of *Postn* with shRNA significantly increased myogenesis levels in DM1 myoblasts, as characterized by elevated myotube area and fusion index values that were close to those of the normal control group (C2C12 GFP-CUG5), and increased expression levels of MRFs and fusion markers. As POSTN is a secreted protein, we considered whether POSTN in the extracellular microenvironment mediated the myogenesis defects in DM1. We treated DM1 myoblast cells with a POSTN-neutralizing antibody and found that this antibody treatment successfully rescued the myogenesis defects, indicating that POSTN in the microenvironment is at least partially responsible for the defective myogenesis in DM1. In line with our findings here, a previous study demonstrated that a POSTN-neutralizing antibody promoted recovery from muscle injuries (Hara et al., 2018). Combined with the finding that *Postn* was significantly upregulated in skeletal muscle of DM1 patients, these results suggest that targeting extracellular POSTN – for example, using neutralizing antibodies – is a potential approach for treating muscle wasting in DM1. This can be an alternative approach to strengthen myogenesis in addition to previously reported therapeutic strategies against muscular dystrophies, such as stem cell transplantation, the inhibition of myostatin, and IGF-1 supplementation (Shavlakadze et al., 2004; Bo Li et al., 2012; Fakhfakh et al., 2012).

Next, we studied how *Postn* regulated the myogenesis process in DM1. Many studies have revealed that *Postn* had crosstalk with the TGF-β/Smad pathway (Blanchard et al., 2008; Lorts et al., 2012; Noguchi et al., 2016; Mitamura et al., 2018; Yue et al., 2021). TGF-β involved pathway was discovered to inhibit myogenesis and promote myoblast proliferation (Massague et al., 1986; Ge et al., 2011). *Smad3* rather than *Smad2* was responsible for the inhibition of TGF-β on the myogenesis process (Liu et al., 2001). Moreover, TGF-β1 and TGF-β2 were found to be upregulated in DM1 patients and associated with arrhythmia and sudden death (Turillazzi et al., 2013). We here tested the levels of SMAD3 and p-SMAD3 in various myoblast differentiation sets. SMAD3 and p-SMAD3 were significantly upregulated in DM1 myoblast cells. When inhibiting *Postn* using an shRNA or a neutralizing antibody, SMAD3 and p-SMAD3 were significantly downregulated. These results strongly suggested that *Postn* might regulate the myogenesis process in DM1 through the TGF-β/Smad3 pathway. This was in line with a previous study that *Postn* was upregulated in muscular dystrophy and its knockout improved muscle structure and function in the muscular dystrophy mouse model via the TGF-β pathway (Lorts et al., 2012). It was also noticeable that the TGF-β/Smad3 pathway was highly activated in both normal and DM1 myoblasts at differentiation day 0, which was consistent with the previous reports that the activation of TGF-β involved pathway inhibited differentiation but induced quiescence of myoblasts (Rathbone et al., 2011). As to *Postn* knockdown in DM1 myoblasts, both SMAD3 and p-SMAD3 were significantly repressed at differentiation day 0 besides day 4, whereas *Postn* showed a significant upregulation in DM1 myoblasts at differentiation day 4 rather than day 0. This conflicting result implied that there might be complicated underlying mechanisms of *Postn* and the TGF-β/Smad3 pathway in undifferentiated myoblasts.

Moreover, the expression of *Postn* gradually increased during *in vitro* myoblast differentiation in both the normal and DM1 groups; this trend was similar to that of many important myogenesis-facilitating factors (Panda et al., 2014; Lee et al., 2017; Horibata et al., 2020). However, this was contrary to the finding that *Postn* downregulation promoted the myogenesis process in both normal and DM1 myoblasts. Meanwhile, there was no significant difference in *Postn* expression levels between the normal and DM1 groups until day 4. However, when *Postn* was downregulated in myoblast cells of both the normal and DM1 groups using shRNA and a neutralizing antibody, myogenesis and fusion markers (*MyoD*, *MyoG*, *Mef2C*, *Mrf4*, *Myomaker*, and *Myomixer*) displayed significant differences earlier than day 4. According to previous reports, *Postn* is upregulated during the regeneration process following muscle injury and disease, suggesting a possible role of *Postn* in myoblast activation. Based on these conflicting findings, we propose a hypothesis: on the one hand, *Postn* is an important factor for myoblast maintenance and its downregulation promotes myoblast activation and differentiation; on the other hand, *Postn* must be upregulated during myoblast differentiation to maintain undifferentiated myoblasts during and after myogenesis. This hypothesis is consistent with the established role of *POSTN* in maintaining cancer stem cells (Malanchi et al., 2011) and warrants further investigation in the future.

## Data Availability Statement

The datasets presented in this study can be found in online repositories. The names of the repository/repositories and accession number(s) can be found below: https://www.ncbi.nlm.nih.gov/geo/query/acc.cgi?acc=GSE174119, accession: GSE174119.

## Author Contributions

XS, ZL, and FX: conceptualization. XS, ZL, and JZ: methodology and software. XS, ZL, CW, FX, JZ, ML, YL, AW, CB, and GZ: investigation. XS and ZL: writing – original draft. XS and GZ: writing – review and editing, and supervision. ZL: visualization. XS: project administration. XS, AW, and CB: funding acquisition.

## Conflict of Interest

The authors declare that the research was conducted in the absence of any commercial or financial relationships that could be construed as a potential conflict of interest.

## Publisher’s Note

All claims expressed in this article are solely those of the authors and do not necessarily represent those of their affiliated organizations, or those of the publisher, the editors and the reviewers. Any product that may be evaluated in this article, or claim that may be made by its manufacturer, is not guaranteed or endorsed by the publisher.

## Footnotes

1
http://dmseq.org/
