# Supplementary material for: Inhibition of Postn Rescues Myogenesis Defects in Myotonic Dystrophy Type 1 Myoblast Model
Source: Front Cell Dev Biol. 2021 Aug 19;9:710112. doi: 10.3389/fcell.2021.710112 (PMC8417118; doi:10.3389/fcell.2021.710112)
Supplement: Supplementary file 1 [file Table_1.DOCX]

**Table S1. The sequences of shRNAs and RT-qPCR primers**

| Name | Sequence |
| --- | --- |
| Scramble-s | TGGGTGAACTCACGTCAGAATTCAAGAGATTCTGACGTGAGTTCACCCTTTTTTC |
| Scramble-as | TCGAGAAAAAAGGGTGAACTCACGTCAGAATCTCTTGAATTCTGACGTGAGTTCACCCA |
| shPostn-s | TGGCAGTCTTCAGCCTATTATTCAAGAGATAATAGGCTGAAGACTGCCTTTTTTC |
| shPostn-as | TCGAGAAAAAAGGCAGTCTTCAGCCTATTATCTCTTGAATAATAGGCTGAAGACTGCCA |
| shMbnl1 -s | TGCACAGAGTTATGCATTAATTCAAGAGATTAATGCATAACTCTGTGCTTTTTTC |
| shMbnl1 -as | TCGAGAAAAAAGCACAGAGTTATGCATTAATCTCTTGAATTAATGCATAACTCTGTGCA |
| Postn-F | CCTGCCCTTATATGCTCTGCT |
| Postn-R | AAACATGGTCAATAGGCATCACT |
| GAPDH-F | CAAGCTCATTTCCTGGTATGACAA |
| GAPDH-R | GGGATAGGGCCTCTCTTGCT |
| MyoD-F | AGTGAATGAGGCCTTCGAGA |
| MyoD-R | GCATCTGAGTCGCCACTGTA |
| MyoG-F | ACTCCCTTACGTCCATCGTG |
| MyoG-R | CAGGACAGCCCCACTTAAAA |
| Mef2C-F | CGGTGTCGTCAGTTGTATGG |
| Mef2C-R | TGCAGTAGATATGCGGCTTG |
| Myomixer-F | CTGAGCAGTTCTGACTGGTG |
| Myomixer-R | CACCATCGGGAGCAATGGAA |
| Myomaker-F | ATCGCTACCAAGAGGCGTT |
| Myomaker-R | CACAGCACAGACAAACCAGG |
| Mrf4-F | ACTGCTAAGGAAGGAGGAGCA |
| Mrf4--R | CTGGCATCTGTGAGCCCTAA |
